# Extracellular Matrix Viscoelasticity Regulates Mammary Branching Morphogenesis

**DOI:** 10.1002/advs.202512873

**Published:** 2025-11-10

**Authors:** Daniella I. Walter, Juliette W. Moore, Abhishek Sharma, Ryan S. Stowers

**Affiliations:** ^1^ Department of Mechanical Engineering University of California Santa Barbara CA 93117 USA; ^2^ Department of Molecular Cellular, and Developmental Biology University of California Santa Barbara CA 93117 USA; ^3^ Department of Bioengineering University of California Santa Barbara CA 93117 USA

**Keywords:** branch elongation, hydrogel, mechanotransduction, stress relaxation, viscoelasticity

## Abstract

Structural and mechanical cues from the extracellular matrix (ECM) regulate tissue morphogenesis. Tissue development has conventionally been studied with ex vivo systems where the mechanical properties of the extracellular environment are either poorly controlled in space and time, lack tunability, or do not mimic ECM mechanics. For these reasons, it remains unknown how matrix stress relaxation rate, a time‐dependent mechanical property that influences several cellular processes, regulates mammary branching morphogenesis. Here, the influence of matrix stress relaxation on mammary branching morphogenesis is systematically investigated using 3D alginate‐collagen matrices and spheroids of human mammary epithelial cells. Slow stress relaxing matrices enhanced branching, which is accompanied by local collagen fiber alignment, intermittent pulling contractions applied to the ECM, and focal adhesion signaling. On the contrary, it is observed that growing spheroids in fast stress relaxing matrices applied isotropic pushing forces to the ECM. Pharmacological inhibition of both Rac1 and non‐muscle myosin II prevented epithelial branch formation, regardless of matrix stress relaxation rate. Interestingly, restricting cellular expansion via increased osmotic pressure is sufficient to impede epithelial branching in slow stress relaxing matrices. This work highlights the importance of stress relaxation in regulating and directing mammary branch elongation.

## Graphical abstract

This study shows that mammary branching morphogenesis is regulated by extracellular matrix (ECM) stress relaxation. Slow stress‐relaxing matrices promote branching through focal adhesion signaling, collagen fiber alignment, and cell contractions, whereas fast stress‐relaxing matrices impair branching and collagen alignment. These findings suggest matrix viscoelasticity can be a key regulator of tissue patterning and development.

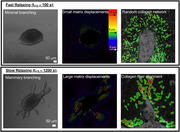

## 1 Introduction

Branching morphogenesis is a complex, multicellular process where cells self‐organize into numerous types of glandular tissue, including mammary glands, lungs, and kidneys. This process is accompanied by remodeling of the extracellular matrix (ECM) architecture.^[^ ^1^, ^2^ ^]^ Much of our current understanding of branching morphogenesis comes from investigations into the effects of biochemical morphogens^[^ ^2^, ^3^, ^4^, ^5^ ^]^ and transcriptional programs,^[^ ^6^, ^7^, ^8^ ^]^ though recent work has revealed that the mechanics of the extracellular matrix can also regulate morphogenetic events.^[^ ^9^, ^10^, ^11^ ^]^ Changes in ECM mechanics can influence a broad array of cellular processes, including differentiation, migration, proliferation, and morphological changes.^[^ ^12^, ^13^, ^14^ ^]^ Within the context of tissue morphogenesis, properties of the ECM, such as matrix stiffness, fiber alignment, pore size, anisotropy, and ECM‐bound cytokines, contribute to tissue patterning and organization throughout the developmental stages.^[^ ^15^, ^16^ ^]^ Despite this, delineating the structural, mechanical, and molecular contributions of the ECM in guiding branch initiation and elongation within mammary epithelium remains challenging due to the dynamic and heterogeneous nature of the ECM.

Engineered extracellular matrices have been pivotal in isolating the role of mechanics and identifying processes that may guide mammary branching, as they enable precise and independent control of specific signals in both time and space, which is something that is difficult to achieve in vivo. For example, 2D in vitro studies have demonstrated that varying matrix stiffness changes the extent of branch initiation within mammary explants in engineered collagen I matrices.^[^ ^17^ ^]^ Additionally, branch elongation and cell migration are guided by tensile forces that drive local fiber alignment.^[^ ^18^, ^19^ ^]^ The ECM protein fibronectin is critical in driving cleft formation in epithelial branching in situ,^[^ ^20^ ^]^ and local accumulation of type I collagen at branch flanks and clefts regulates mammary branching ex vivo.^[^ ^21^ ^]^ This demonstrates how ECM dynamics, both ex vivo and in situ, appear to modulate local mechanics similarly to what has been previously observed in vitro.^[^ ^17^, ^18^, ^19^ ^]^ Collectively, these studies highlight the critical role of ECM mechanics in mammary branching.

While it is known that matrix stiffness regulates branching morphogenesis,^[^ ^22^, ^23^, ^24^ ^]^ the effect of ECM viscoelasticity remains an open question. Biological tissues are viscoelastic,^[^ ^25^, ^26^ ^]^ meaning they exhibit both elastic solid‐like behavior and viscous fluid‐like behavior. In response to a constant deformation, viscoelastic materials will relax stress over time, while for purely elastic materials, stress will remain constant over time. ECM viscoelasticity has been shown to influence cell spreading,^[^ ^27^, ^28^ ^]^ proliferation,^[^ ^28^, ^29^ ^]^ migration,^[^ ^30^, ^31^ ^]^ and differentiation.^[^ ^32^, ^33^, ^34^ ^]^ Very recently, ECM viscoelasticity has been shown to influence intestinal crypt morphogenesis, breast epithelial cell growth, and nephrogenesis in kidney organoids.^[^ ^35^, ^36^, ^37^ ^]^ However, it remains unknown whether matrix viscoelasticity influences branching morphogenesis generally, including within mammary tissue, and the mechanisms underlying such processes are yet to be determined.

Here, we report the results of a systematic investigation into the role of matrix stress relaxation in regulating branching of human mammary epithelium. We explored biophysical mechanisms underlying mammary branch development and identified signaling pathways that are differentially regulated via matrix stress relaxation rate. This work reveals how matrix viscoelasticity regulates mammary branching, which can be leveraged for applications in tissue engineering and regenerative medicine, and may be relevant in other branching morphogenesis contexts as well.

## 2 Results

### 2.1 Slower Matrix Stress Relaxation Enhances Mammary Branching

**Figure 1:**
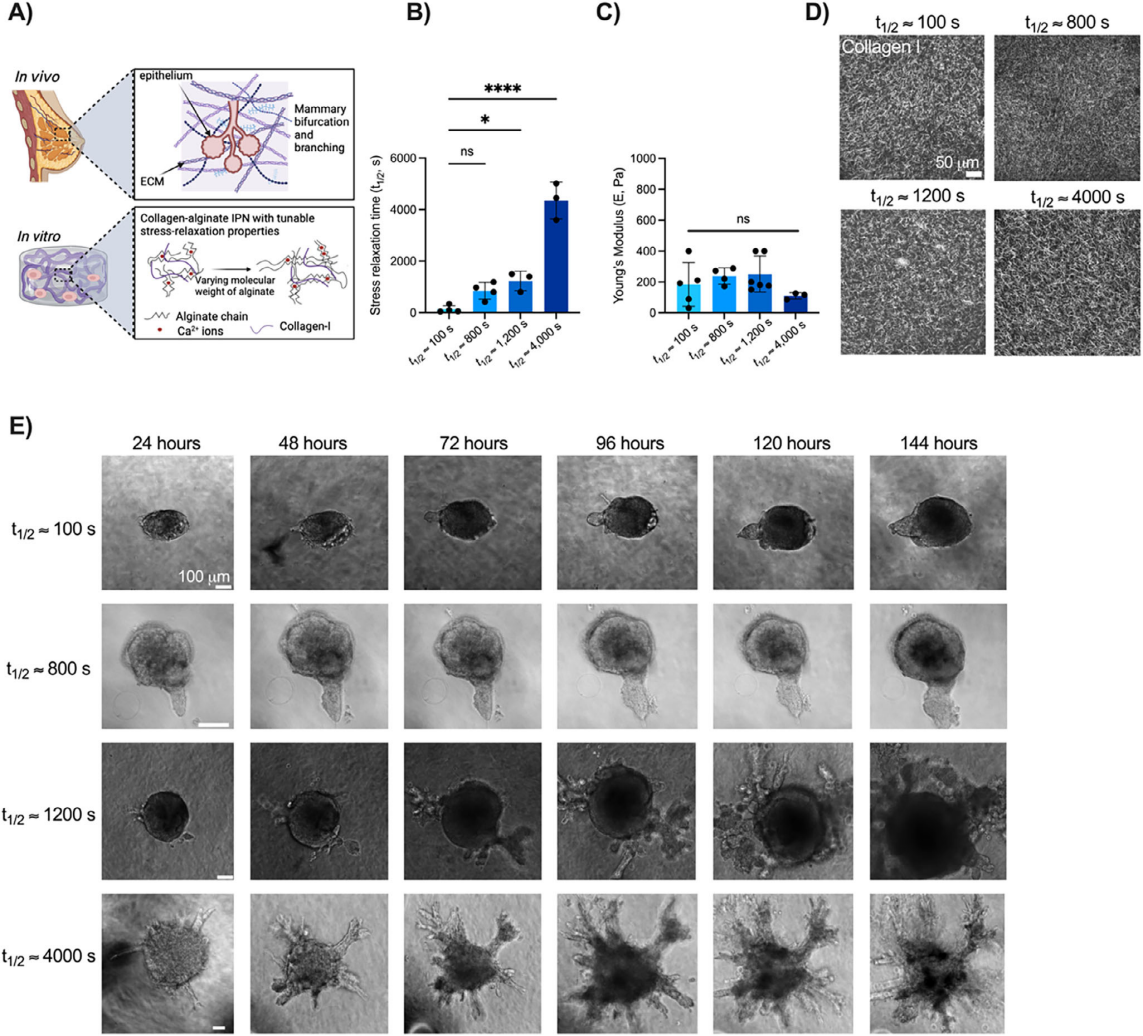
Matrix viscoelasticity regulates mammary branching of human epithelial spheroids. a) Schematic depicting how mammary gland development is influenced by mechanical and biochemical cues within the in vivo microenvironment. 3D alginate‐collagen interpenetrating networks (IPNs) enable independent tuning of the viscoelasticity of the matrix in vitro. Image created with BioRender.com. b) Alginates of different molecular weights can be used to fabricate matrices with varying stress relaxation times. c) Time sweep revealed that hydrogels with varying stress relaxation times had Young's moduli that were not significantly different. d) Representative confocal reflectance microscopy images depict collagen microarchitectures of matrices with varying stress relaxation times. e) Brightfield images of MCF10A spheroids in 3D slow stress‐relaxing matrices (t_1/2 _ ≈ 1200 s, t_1/2_ ≈ 4000 s) and fast stress‐relaxing matrices (t_1/2_ ≈ 100 s, t_1/2_ = 800 s) over 6 days.  All data are represented as mean ± standard deviation (SD). Statistical significance was determined using one‐way ANOVA with Dunnett's multiple comparison tests. *n* = 5–15 images per replicate, with three independent replicates. Significance is indicated as follows: ^****^ *p* < 0001, ^*^ *p* < 05, and n.s. = not significant.

We used tunable viscoelastic matrices to probe the impact of different stress relaxation rates on mammary branching. We formed 3D matrices using interpenetrating network (IPN) hydrogels composed of alginate and collagen I. Alginate is a bioinert polysaccharide that enables viscoelasticity to be tuned independently of stiffness by varying its molecular weight.^[^ ^38^ ^]^ Divalent cations, such as calcium, form ionic crosslinks between alginate chains, which can break under stress and reform, allowing local matrix flow and giving rise to the stress‐relaxing properties of the hydrogel^[^ ^39^ ^]^ (**Figure** **1**A). Collagen I was incorporated as it is the major structural protein within the mammary gland^[^ ^15^ ^]^ and has been previously shown to induce elongation in mammary epithelial cells.^[^ ^40^ ^]^ The stress‐relaxing properties of healthy mammary gland tissue are not well‐characterized, so we fabricated hydrogels within a range of stress relaxation half times where cells are mechanically responsive^[^ ^26^ ^]^ (*t*_1/2_ ≈ 100, 800, 1200, and 4000 s) (Figure 1B) by using alginates of different molecular weights. The calcium concentration for alginate crosslinking was optimized to achieve a constant elastic modulus of ≈200 Pa across all stress relaxation groups (Figure 1C), mimicking physiological mammary tissue.^[^ ^41^ ^]^ Importantly, we were able to tune the mechanical properties of the hydrogel independently of the collagen microarchitecture of the substrates (Figure 1D).

**Figure 2:**
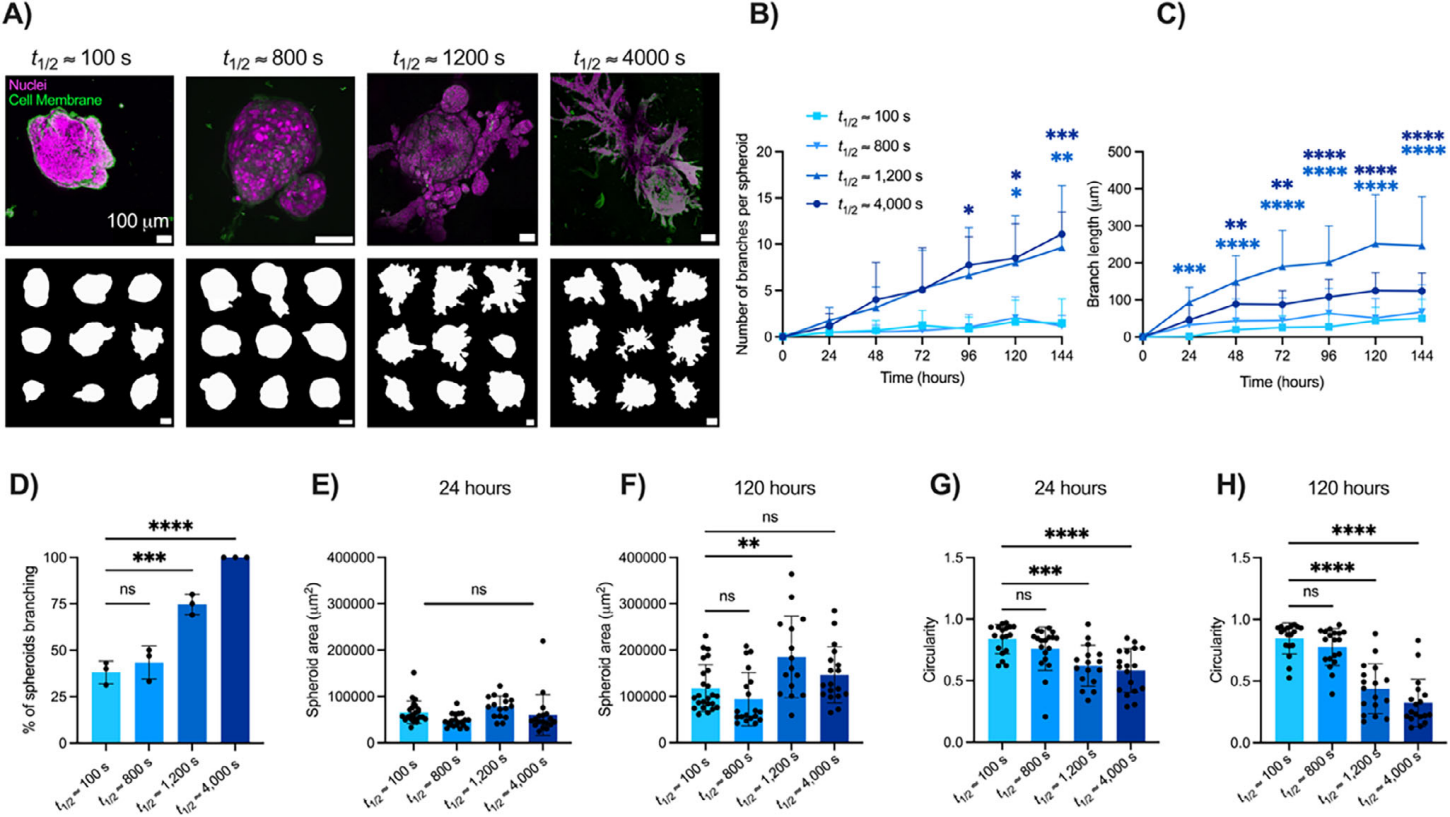
Mammary epithelial spheroid morphology and branch formation vary with stress relaxation rate. a) Confocal images of an MCF10A spheroid in slow stress‐relaxing matrices (t_1/2 _ ≈ 1200 s, t_1/2_ ≈ 4000 s) and fast stress‐relaxing matrices (t_1/2_ ≈ 100 s, t_1/2_ = 800 s). Cell membranes were labeled with Rhodamine B (green) and the nuclei with Hoechst 33342 (magenta). Representative outlines of MCF10A spheroid morphologies are portrayed after 120 h in culture in varying stress‐relaxing matrices. b) Quantification of branches revealed that more branches were produced in slow stress‐relaxing matrices (t_1/2 _ ≈ 1200 s, t_1/2_ ≈ 4000 s) and c) this was accompanied by greater branch lengths. d) Branch frequency was significantly enhanced in slow stress‐relaxing matrices (t_1/2 _ ≈ 1200 s, t_1/2_ ≈ 4000 s), where each data point represents the percentage of spheroids that branched in an independent experiment, and quantified on day 7. e) Quantification of the cross‐sectional area of MCF10A spheroids at 24 h and f) 120 h. g) Quantification of circularity at 24 h and h) 120 h in slow and fast stress‐relaxing matrices. Statistical significance was determined using one‐way ANOVA with Šídák's or Dunnett's multiple comparison tests: ^****^ *p* < 0001, ^***^ *p* < 001, ^**^ *p* < 01, ^*^ *p* < 05, and n.s. = not significant, and performed with respect to the fast stress‐relaxing condition (t_1/2 _ ≈ 100 s). *n* = 5–15 images per replicate, with three independent replicates. All data are represented as mean ± SD.

We next assessed the influence of matrix stress relaxation on mammary branching. MCF10A human mammary epithelial cells, a cell line commonly used to model mammary development,^[^ ^42^ ^]^ were formed into spheroids of ≈3000 cells. MCF10A spheroids were encapsulated in alginate‐collagen matrices with various stress relaxation half times (*t*_1/2_ ≈ 100 s, *t*_1/2_ ≈ 800 s, *t*_1/2_ ≈ 1200 s, and *t*_1/2_ ≈ 4000 s). Over the course of six days, tissues in fast stress‐relaxing conditions (*t*_1/2_ ≈ 100 and 800 s) expanded isotropically and formed buds into the surrounding matrix (Figure 1E). However, spheroids in slower stress‐relaxing conditions (*t*_1/2_ ≈ 1200 and 4000 s) broke symmetry and branched extensively (Figure 1E). Using confocal microscopy and fluorescently labeled cells, we observed compact and symmetric geometries of spheroids in fast stress‐relaxing matrices, but several branched structures stemming from the spheroid body in the slow stress‐relaxing matrices (**Figure** **2**A). This was characterized by a significant increase (586%) in the total number of branches (Figure 2B) and a significant increase in branch length (390%) within slow stress‐relaxing matrices (*t*_1/2_ ≈ 1200 s) compared to fast stress‐relaxing matrices (*t*_1/2_ ≈ 100 s) after six days in culture (Figure 2C). To account for experimental heterogeneity, we quantified the percentage of spheroids branching in any given experiment and found that spheroids in slow stress‐relaxing matrices exhibited the greatest branch frequency when compared to spheroids in fast stress‐relaxing matrices (Figure 2D). Additionally, the cross‐sectional area of the spheroid was not significantly different between stress relaxation groups after 24 h in culture (Figure 2E), but there was a significant increase in cross‐sectional area (57%) after 120 h in slow stress‐relaxing matrices (*t*_1/2_ ≈ 1200 s) (Figure 2F). Spheroids in slow stress‐relaxing matrices were also significantly less circular than those in fast stress‐relaxing matrices, both at 24 and 120 h (Figure 2G,H). We also sought to achieve slow stress‐relaxing matrices composed only of collagen by covalent crosslinking with tissue transglutaminase (tTG). Mechanical characterization of unmodified collagen‐only gels revealed that they were significantly softer than fast stress‐relaxing alginate–collagen IPNs (Figure S1, Supporting Information). We found that covalent crosslinking of collagen matrices with transglutaminase (tTG) increased both the Young's modulus and stress relaxation time compared to untreated matrices (Figure S2A,B, Supporting Information). However, we did not observe branching in tTG‐collagen matrices, and instead mammary epithelial spheroid growth was restricted (Figure S2C, Supporting Information). This observation is consistent with prior work showing that covalently crosslinked matrices restrict cell spreading,^[^ ^43^ ^]^ as it reduces the ability of cells to plastically remodel the collagen network.^[^ ^44^ ^]^ Our results demonstrate that matrix stress relaxation plays a key role in regulating mammary epithelial spheroid morphology and branch formation.

### 2.2 Slow Stress‐Relaxing Matrices Promote Collagen Fiber Alignment Along the Branching Axis

**Figure 3:**
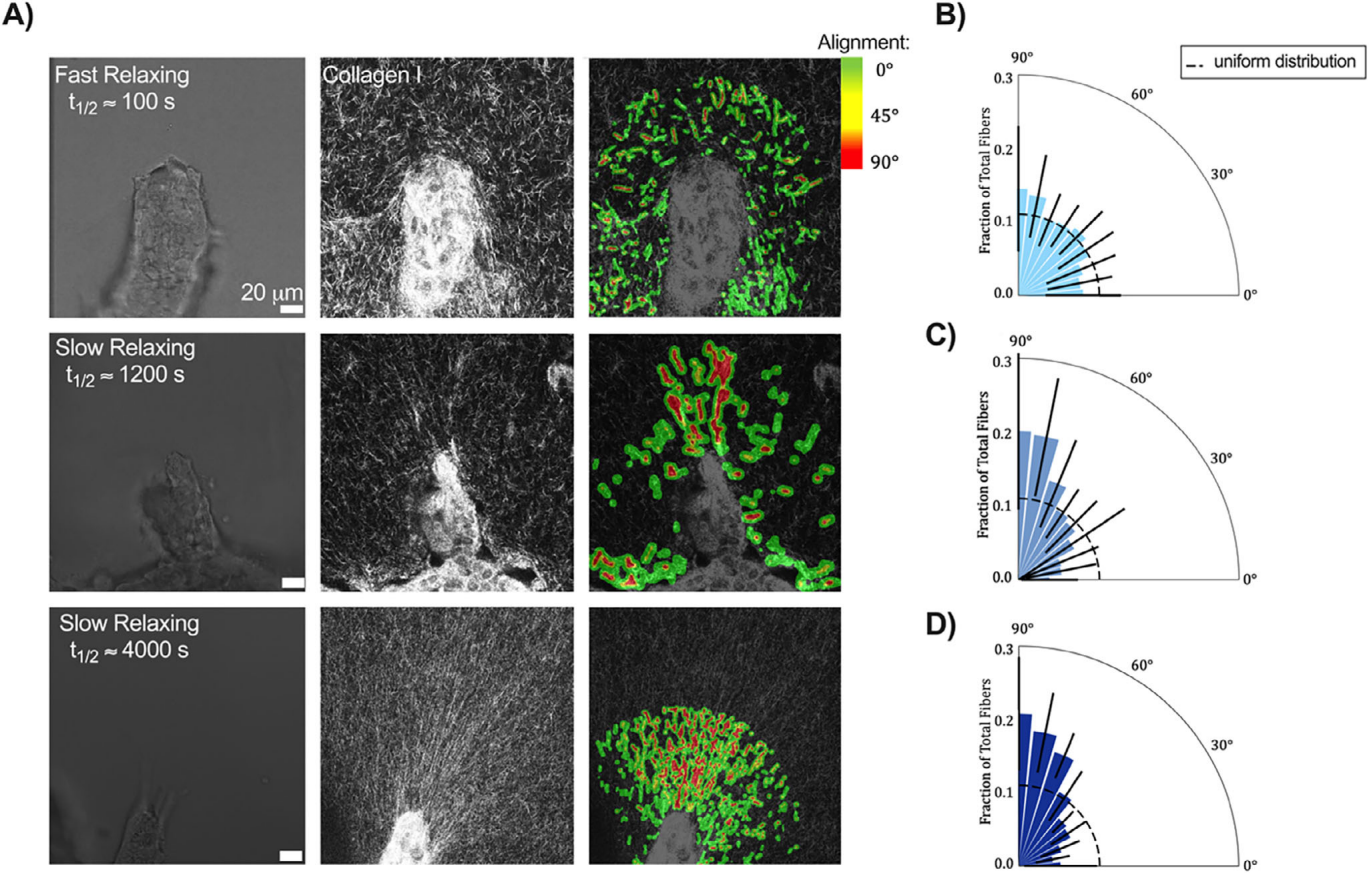
Slow relaxing matrices promote collagen fiber alignment along the branching axis. a) Representative brightfield and confocal reflectance images of the collagen fiber network surrounding an MCF10A spheroid branch in various stress‐relaxing conditions (t_1/2 _ ≈ 100 s, t_1/2 _ ≈ 1200 s, t_1/2_ ≈ 4000 s) after 96 h in culture. The heatmap depicts the extent of collagen fiber alignment via *CurveAlign*.  b) Quantification of the relative orientation angle along the branching axis demonstrated that collagen fibers are randomly aligned in the t_1/2_ ≈ 100 s gel, c) highly aligned in the t_1/2 _ ≈ 1200 s gel, and d) also highly aligned in the t_1/2_ ≈ 4000 s gel. The dashed line represents the expected value if all fibers were uniformly distributed within the bins. *n* = 9 images per replicate, with three independent replicates. Black error bars indicate SD for each group. Kruskal–Wallis with Dunn's post hoc multiple comparison tests was used to compare the fraction of fibers in each bin in C and D with the corresponding bin in B. ^**^ *p* < 01 between all groups with angles greater than 70*°*.

We next sought to elucidate the biophysical mechanisms through which epithelium can generate forces to elongate branches and pattern epithelial trees. Previous work has found that collagen can accumulate around the outer flanks of mammary branches and at branch bifurcation sites.^[^ ^21^ ^]^ Further, mammary gland explants will branch along a pre‐aligned collagen network, following the direction of the collagen orientation axis.^[^ ^45^ ^]^ To determine if viscoelasticity can drive mammary morphogenesis via changes in collagen fiber alignment, we employed confocal reflectance microscopy to visualize the collagen network surrounding epithelial branches in both fast (*t*_1/2_ ≈ 100 s) and slow stress‐relaxing matrices (*t*_1/2_ ≈ 1200 s, 4000 s). We then calculated the orientation angle (0°–90°) between the spheroid boundary and local collagen fibers. Regions of interest were drawn to delineate collagen fiber alignment along the branching axis and adjacent to the branching axis.^[^ ^40^ ^]^ Confocal reflectance images and a density heat map demonstrated enhanced collagen fiber alignment in slow stress‐relaxing matrices along the branching axis (**Figure** **3**A). Upon binning and normalizing the orientation values, we observed significantly increased collagen fiber alignment (corresponding to bins between 60*°* and 90*°*) in front of the branching axis in slow stress‐relaxing matrices, but not in fast stress‐relaxing matrices (Figure 3B–D). The collagen fiber orientation values adjacent to the mammary branches (away from the tip) were uniformly distributed regardless of matrix stress relaxation (Figure S3A–C, Supporting Information), indicating a random collagen fiber network. Thus, our findings suggest that mammary epithelial cells spatially remodel their collagen matrix to promote branching morphogenesis in a stress relaxation‐dependent manner.

### 2.3 Cell‐Generated Forces Displace the Matrix to Facilitate Mammary Branching in Slow Stress‐Relaxing Matrices

**Figure 4:**
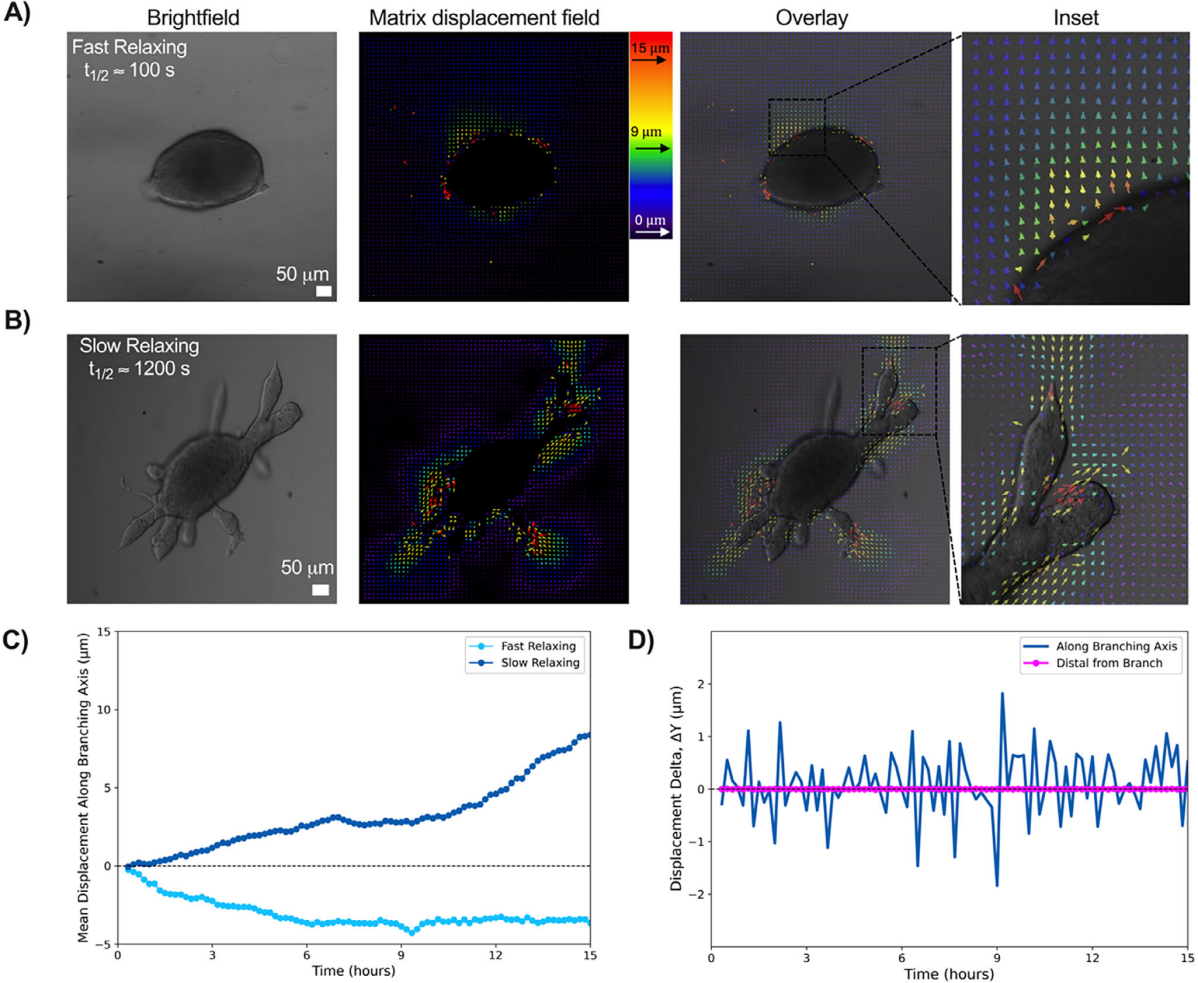
Cell‐generated non‐continuous contractions displace the matrix to facilitate mammary branching in slow stress‐relaxing matrices. a) Matrix displacement field of an MCF10A spheroid in a fast stress‐relaxing matrix demonstrated minimal displacements were applied. b) Matrix displacement field of an MCF10A spheroid in a slow stress‐relaxing matrix demonstrated that high matrix displacements were applied in front of branches. Matrix displacement field generated via particle image velocimetry (PIV) Image J plugin. c) Positive cumulative mean displacements of greater magnitude were observed in slow stress‐relaxing matrices, suggesting pulling forces, while fast stress‐relaxing matrices showed smaller, negative displacements, suggesting pushing forces. d) Quantification of mean displacement delta demonstrated that mammary epithelium exerts non‐continuous contractions to extend their mammary branches in slow stress‐relaxing matrices. *n* = 5 videos analyzed per replicate, with three independent replicates. Bead displacement position data acquired from *Imaris* and analyzed and plotted in MATLAB.

Since we observed enhanced collagen fiber alignment in front of the branches in slow stress‐relaxing matrices, we next evaluated the extent of matrix deformation during branching. We encapsulated MCF10A spheroids in both slow (*t*_1/2_ ≈ 1200 s) and fast (*t*_1/2_ ≈ 100 s) stress‐relaxing conditions, along with 0.2 µm fluorescent beads for 48 h, and tracked the bead displacement over the next 16 h in culture (**Figure** **4**A,B). Spheroids in fast stress‐relaxing conditions generated small, isotropic pushing displacements in the surrounding matrix (Figure 4A; Video S1, Supporting Information). Interestingly, branch tips in slow stress‐relaxing matrices generated displacements in the pulling direction during branch extension, whereas branch bifurcation sites exhibited pushing displacements (Figure 4B; Video S2, Supporting Information). This demonstrates how cells spatially pattern the application of force to the ECM to enable branch extension and bifurcation. We next quantified the direction and magnitude of displacements with respect to a reference frame where the y‐axis was oriented toward the branch, so that any displacements applied directionally toward the branch would result in a positive y‐axis displacement, and any displacements away from the branch would result in a negative *y*‐axis displacement. Interestingly, we found that larger cumulative displacements occurred in front of branch tips in slow stress‐relaxing matrices, compared to their fast stress‐relaxing counterparts (Figure 4C). We observed that the displacements were both positive and of greater magnitude in slow stress‐relaxing matrices than in fast stress‐relaxing matrices (Figure 4C). This indicates that mammary epithelium exerts pulling forces (as demonstrated by the positive displacement field) to extend branches in slow stress‐relaxing matrices, but in fast stress‐relaxing matrices, spheroids exert pushing forces (as demonstrated by the negative displacement field). Collectively, these results demonstrate how the cell‐generated forces that drive tissue growth and branching are dependent on the stress relaxation of the matrix. We next looked at the changes in displacement between consecutive frames (delta displacement) in front of a representative branch within a slow stress‐relaxing matrix. We found an intermittent increase and decrease over time, compared to a region of interest distal from the branch, where there was no change in delta displacement over time (Figure 4D). This illustrates that cells exert pulling forces in an intermittent, non‐continuous manner in slow stress‐relaxing matrices, and these forces are applied in front of elongating branches.

### 2.4 Actomyosin Contractility and Phosphorylated Focal Adhesion Kinase Contribute to Mammary Branch Elongation

**Figure 5:**
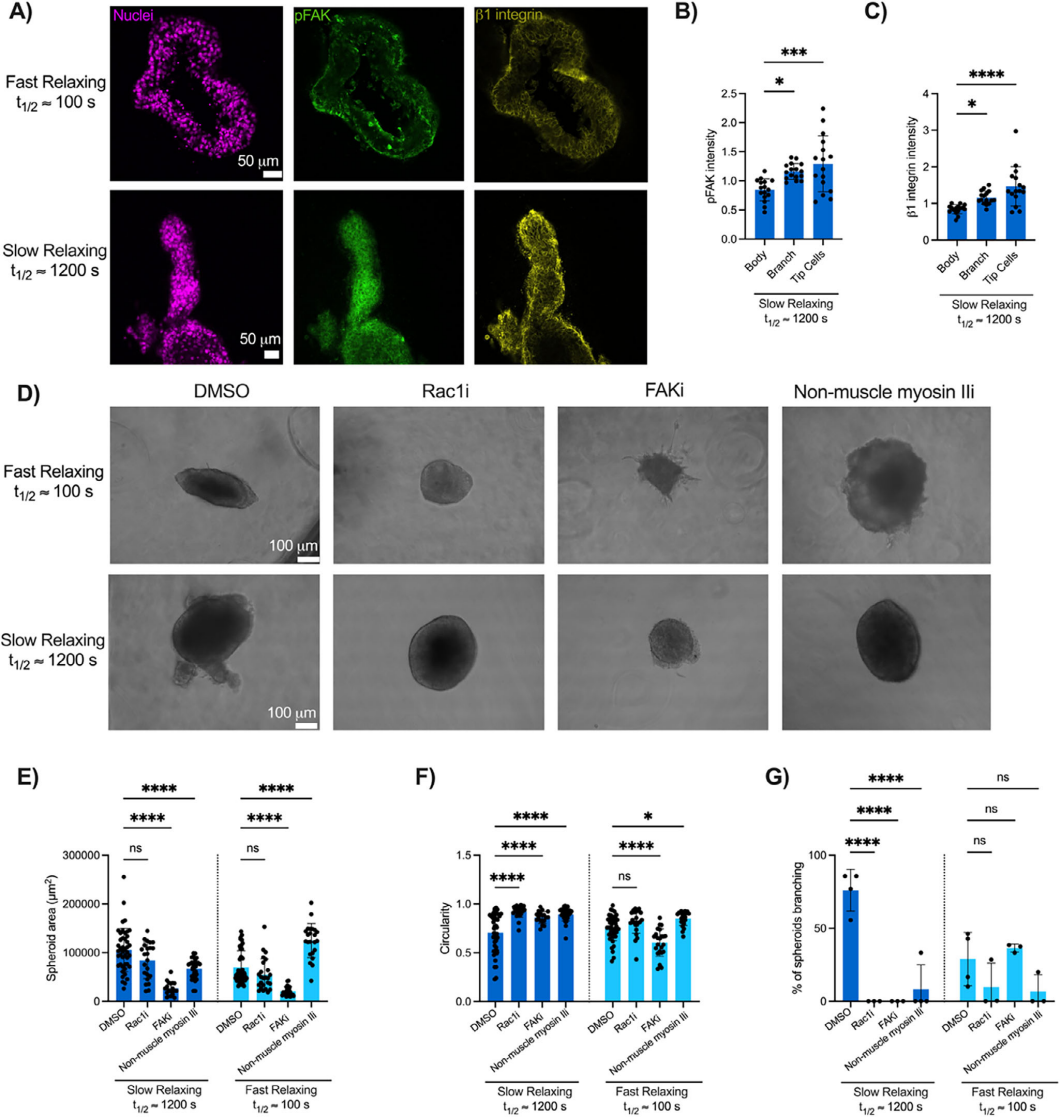
Actomyosin contractility and phosphorylated focal adhesion kinase contribute to mammary branch elongation. a) Immunohistochemical stains of phosphorylated FAK (Tyr397) and β1 integrin in MCF10A spheroids in both fast (t_1/2_ ≈ 100 s) and slow (t_1/2_ ≈ 1200 s) and stress‐relaxing matrices after 7 days in culture. b) There was a greater abundance of both FAK phosphorylation (pFAK) and c) β1 integrin at the tip cells of a mammary duct compared to the spheroid body in slow stress‐relaxing gels. Image intensity values were normalized to the nuclei stain (Hoechst 33342). d) Representative brightfield images of MCF10A spheroids cultured in fast and slow stress‐relaxing gels after 7 days with the treatment of Rac1, FAK, or non‐muscle myosin II inhibitors, or DMSO as a vehicle control. e) Quantification of spheroid area, f) circularity, and g) branch frequency when cultured in the indicated conditions. Statistical significance was determined using one‐way ANOVA and with Tukey's or Šídák's multiple comparison tests: ^****^ *p* < 0001, ^***^ *p* < 001, ^**^ *p* < 01, ^*^ *p* < 05, and n.s. = not significant.  *n* = 5–15 images per replicate, with three independent replicates. All data are represented as mean ± SD.

Given that we observed enhanced collagen fiber alignment at the leading edge of elongating mammary branches, and that mechanical forces are sufficient to orient and align collagen fibers,^[^ ^46^ ^]^ we hypothesized that cell contractility drives collagen fiber alignment, and thereby mammary epithelial branch elongation. We investigated the role of β1 integrin, focal adhesion kinase activity, and actomyosin‐based contractility on mammary branching, as these are key components of mechanoresponsive pathways that enable cellular force generation. Integrin binding could enable force transmission to align collagen fibers, and this would be associated with downstream signaling proteins, including focal adhesion kinase (FAK),^[^ ^47^ ^]^ which has been shown to mediate tissue responses to ECM remodeling.^[^ ^48^ ^]^ In particular, we examined β1 integrins, as they are known to play a role in collagen fiber bundling^[^ ^49^ ^]^ and are implicated in mammary branching.^[^ ^50^ ^]^ An examination of the localization of β1 integrin and phosphorylated FAK (Tyr397) via immunostaining and confocal microscopy revealed significant increases (74% and 53%, respectively) in the abundance of these proteins at the tip cells of mammary branches in slow stress‐relaxing matrices compared to the abundance in the spheroid body (**Figure** **5**A–C). Next, we investigated the hypothesis that the ability of mammary epithelial cells to undergo branching morphogenesis is dependent on cell contractility and force‐generating pathways. Non‐muscle myosin II is a motor protein that enables cell contractility and mechanical stability, and Rac1, a GTPase that regulates the formation and maintenance of focal adhesions, has been shown to regulate branch elongation and tissue growth.^[^ ^36^, ^51^ ^]^ We pharmacologically inhibited FAK, non‐muscle myosin II, and Rac1, and found that inhibition of FAK led to a significant decrease in spheroid area in both slow and fast stress‐relaxing matrices (75% and 70%, respectively) compared to untreated controls (Figure 5D,E). Inhibition of non‐muscle myosin II led to a significant decrease (37%) in spheroid area in slow stress‐relaxing matrices compared to untreated controls, but a significant increase (78%) in spheroid area in fast stress‐relaxing matrices (Figure 5E). Interestingly, inhibiting Rac1 resulted in no significant effect on spheroid area in slow or fast stress‐relaxing matrices (Figure 5E). In slow stress‐relaxing matrices, circularity was significantly increased when Rac1, FAK, and non‐muscle myosin II were inhibited (31%, 22%, and 27%, respectively), although in fast stress‐relaxing matrices, inhibiting FAK led to a 20% significant decrease in circularity due to the formation of cellular protrusions throughout the spheroid (Figure 5F). Further, inhibition of all three signaling pathways led to a significant decrease in branching frequency in slow stress‐relaxing matrices, but there was no significant difference within fast stress‐relaxing matrices (Figure 5G).

Collectively, these results show that β1 integrin, phosphorylated FAK, Rac1, and non‐muscle myosin II play key roles in contributing to mammary morphogenesis in slow stress‐relaxing matrices.

### 2.5 Hyperosmotic Stress Reduces Mammary Spheroid Growth and Branching in Slow Stress‐Relaxing Matrices

**Figure 6:**
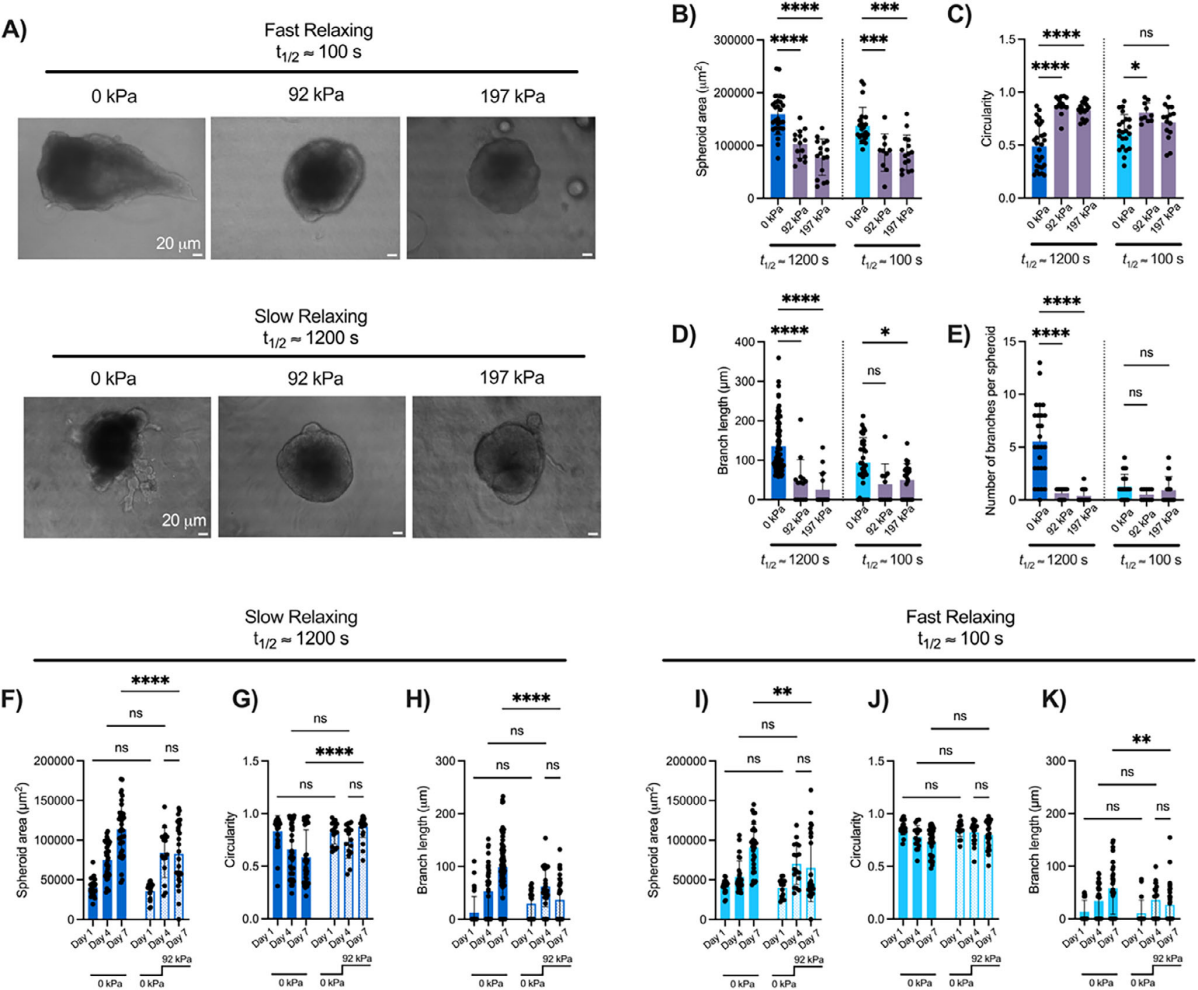
Hyperosmotic stress modulates mammary spheroid growth and branching in slow stress‐relaxing matrices. a) Brightfield images of MCF10A spheroids in various stress‐relaxing conditions under continuous hyperosmotic stress for 7 days. b) Quantification of MCF10A spheroid area under varied osmotic pressure (Δ*P*  =  0, 92, 197 kPa) for 7 days, in both slow (t_1/2_ ≈ 1200 s) and fast (t_1/2_ ≈ 100 s) relaxing matrices. c) Circularity was enhanced in slow stress‐relaxing gels when spheroids were subjected to hypertonic media. d) Quantification of branch length demonstrated that spheroids branched to a lesser extent in slow stress‐relaxing gels under an osmotic pressure of Δ*P*  =  92 and 197 kPa. e) In slow stress‐relaxing gels, spheroids branched less when subjected to hyperosmotic pressure, but there was no significant difference in fast stress‐relaxing gels. f) MCF10A spheroid growth in slow stress‐relaxing matrices was halted when spheroids were subjected to hyperosmotic pressure on day 4 in culture, and this was accompanied by g) increased circularity and h) decreased branch length. i) MCF10A spheroid growth in fast stress‐relaxing matrices was halted when spheroids were subjected to hyperosmotic pressure on day 4 in culture. j) There was no significant difference in circularity when osmotic pressure was applied on day 4 in culture in fast stress‐relaxing matrices, although k) branch length decreased significantly compared to the control. Statistical significance was determined using one‐way ANOVA with Šídák's multiple comparison tests: ^****^ *p* < 0001, ^***^ *p* < 001, ^**^ *p* < 01, ^*^ *p* < 05 and n.s. = not significant. *n* = 5–15 images per replicate, with three independent replicates. All data are represented as mean ± SD.

ECM viscoelasticity relates strongly to the concept of confinement,^[^ ^52^ ^]^ as it dictates the extent to which cells can deform and remodel their microenvironment over time. Thus, we next probed the role of confinement on mammary branching by applying hyperosmotic stress to mammary epithelial spheroids. Previous work has shown that the pressure of fluid within the developing lung can drive morphogenesis^[^ ^53^ ^]^ and that breast epithelial cellular membrane breaching is driven by cell volume expansion.^[^ ^54^ ^]^ Extracellular matrix mechanics impact single cell volume expansion, as recent work has found that A7 cells will decrease their water content in response to an increased substrate stiffness,^[^ ^55^ ^]^ and mesenchymal stem cell (MSC) is regulated by stress relaxation.^[^ ^34^ ^]^ Here, we applied hyperosmotic stress to spheroids by the addition of varying concentrations of 400 Da polyethylene glycol (PEG) in growth media, as in prior studies.^[^ ^55^, ^56^ ^]^ After a week of culture under hyperosmotic pressure, we found that mammary branching was significantly impeded in both slow and fast stress‐relaxing matrices compared to unperturbed controls (**Figure** **6**A). We observed that both low (Δ*P*  =  92 kPa, 37.5 mOsm L^−1^) and high osmotic pressures (Δ*P*  =  197 kPa, 75 mOsm L^−1^) led to a significant decrease in cross‐sectional spheroid area in both slow and fast stress‐relaxing matrices compared to the control (Δ*P*  =  0 kPa, 0 mOsm L^−1^) (Figure 6B). Additionally, low (Δ*P*  =  92 kPa) and high osmotic pressures (Δ*P*  =  197 kPa) significantly enhanced spheroid circularity in slow stress‐relaxing matrices (Figure 6C). Only low osmotic pressure (Δ*P*  =  92 kPa) enhanced circularity in fast stress‐relaxing matrices, although spheroids in fast stress‐relaxing matrices were already very circular (Figure 6C). Furthermore, branch length and branch number were significantly reduced in slow stress‐relaxing matrices under both low (Δ*P*  =  92 kPa) and high (Δ*P*  =  197 kPa) osmotic pressures (Figure 6D,E). Interestingly, we found that applying varying levels of hypoosmotic stress (Δ*P*  =   − 155 kPa, Δ*P*  =   − 310 kPa) for a week in culture was not sufficient to enhance mammary growth or branching in either slow or fast stress‐relaxing matrices (Figure S4, Supporting Information). To further investigate the extent to which hyperosmotic pressure impairs mammary branching, we dynamically modulated the applied pressure within our cell culture system. Spheroids were first allowed to grow for four days in culture under normal conditions, after which hyperosmotic pressure was applied and maintained for the following three days. We found that applying low osmotic pressure (Δ*P*  =  92 kPa) after four days in culture impaired the ability for spheroids to grow and form branches in slow stress‐relaxing matrices for the subsequent three days (Figure 6F–H; Figure S5A, Supporting Information). This trend was also consistent for spheroids cultured in fast stress‐relaxing matrices (Figure 6I–K; Figure S5B, Supporting Information). This effect was further amplified when we applied higher osmotic pressures (Δ*P*  =  197 kPa) after four days in culture, and we found that higher osmotic pressures were sufficient to halt both branching and cellular expansion in both slow and fast stress‐relaxing matrices (Figure S6, Supporting Information). Additionally, we conducted a complementary experiment where osmotic pressure was applied for the first four days in culture, followed by a return to normal conditions for the remaining three days. We found that when the lower osmotic pressure (Δ*P*  =  92 kPa) was released after four days in culture, spheroids were able to grow but failed to resume branching in both slow and fast stress‐relaxing matrices (Figure S7A–H, Supporting Information).

When we performed the same experiment with higher osmotic pressures (Δ*P*  =  197 kPa), we found that spheroids were unable to recover branching in both slow and fast stress‐relaxing matrices after osmotic pressure was released on day four (Figure S7I–P, Supporting Information). In summary, our findings demonstrate that changes in osmotic pressure can disrupt mammary morphogenesis, even after this pressure is subsequently released. This suggests that transient increases in confinement, whether through osmotic pressure or changes in ECM viscoelasticity, play a critical role in regulating mammary epithelial branching.

## 3 Discussion and Conclusion

Here, we provide evidence that matrix stress relaxation is a key parameter in regulating mammary branching morphogenesis. Our unique approach allows us to decouple matrix stress relaxation from matrix stiffness and collagen fiber architecture to better understand how stress relaxation independently governs mammary branching. Our study reveals that cell‐generated forces, driven by actomyosin contractility and focal adhesion kinase activity, facilitate branch elongation in slow stress‐relaxing matrices. Our results suggest that mammary epithelium locally remodels the collagen fiber network and displaces the ECM to enable branch elongation. Importantly, we find that this behavior only emerges in slow stress‐relaxing matrices, thereby demonstrating the importance of the timescale of stress relaxation in tissue morphogenesis.

While the influence of matrix stiffness on tissue morphogenesis has been increasingly recognized,^[^ ^22^, ^23^, ^24^ ^]^ the mechanism of how viscoelasticity mediates morphogenesis remains poorly understood. Here, we show that branching morphogenesis is supported in slow stress‐relaxing matrices, but not in fast stress‐relaxing matrices. Although the stress‐relaxing properties of healthy mammary gland tissue are not well‐characterized, the mammary gland is surrounded by adipose tissue, which exhibits relatively slow stress relaxation, on the order of hundreds of seconds.^[^ ^26^ ^]^ This may, in part, explain why the slow stress‐relaxing matrices promote mammary branching. Prior studies have found that cells migrate collectively via local proteolysis of the matrix or ECM softening,^[^ ^19^, ^57^ ^]^ and our findings raise the possibility that mechanical confinement in slow stress‐relaxing matrices may spatially constrain cells, thereby promoting branch initiation in local regions of heterogeneity. A recent report found that mammary epithelial spheroids undergo symmetry breaking and invade the surrounding matrix when embedded in stiff (5000 Pa) and fast stress‐relaxing alginate matrices.^[^ ^36^ ^]^ We hypothesize that the differences in MCF10A spheroid behavior are attributable to two key distinctions in the microenvironmental properties between these studies; i) the matrices used in our experiments were over an order of magnitude softer (200 Pa vs. 5000 Pa), and ii) we incorporated fibrillar collagen I within the matrix for cell adhesion, instead of a direct conjugation of the RGD adhesion motif to the alginate. These distinctions highlight the critical role of matrix composition and matrix stiffness in regulating cell behavior and morphogenetic processes.

While the role of the extracellular matrix in guiding epithelial form and function has long been recognized, the specific role of collagen I in directing mammary branch extension has been contested. In murine models, prior studies have demonstrated that patterning of collagen fibers in the mammary gland stroma regulates epithelial orientation during development^[^ ^45^ ^]^ and that cell‐induced matrix alignment facilitates collective cellular migration.^[^ ^58^ ^]^ It has been proposed that ECM fibers may serve as conduits for long‐range stress transmission, thereby enabling mechanotransduction across tissue scales.^[^ ^59^ ^]^ Conversely, recent reports suggest that directional epithelial outgrowth occurs independently of collagen I alignment,^[^ ^21^ ^]^ and computational simulations suggest branching can occur in the absence of long‐range guidance cues from the ECM.^[^ ^60^ ^]^ Here, we show that human mammary epithelia apply directional forces to mechanically constrain and stabilize the ECM and guide branch development, which is consistent with recent studies that show that collagen fiber alignment is generated by expanding branches of human mammary organoids.^[^ ^18^ ^]^ While the temporal sequence of these events remains to be fully resolved, our findings support a model of mechanical feedback between epithelial dynamics and ECM organization, which is mediated by the viscoelastic properties of the matrix.

Notably, we have also shown in this study how integrin‐mediated, non‐continuous displacements are applied to the ECM to drive branch elongation. Previous work has suggested the importance of phosphorylated FAK and integrin activity in regulating collagen fiber alignment in stress‐relaxing matrices, particularly within the context of human MSCs.^[^ ^27^ ^]^ Additionally, β1 integrin signaling is required for directional migration during mammary morphogenesis.^[^ ^61^ ^]^ Our work builds upon this foundation by showing that both FAK and Rac1 are critical for mammary branching in slow stress‐relaxing matrices, and that phosphorylated FAK and β1 integrin localize to the tip cells of extending branches. Consistent with this, others have found actin nucleation factors to be important modulators of dynamic cell protrusions in viscoelastic fibrillar ECMs.^[^ ^62^ ^]^

Given the role of integrins in coupling ECM adhesions to the actin cytoskeleton, these findings suggest a mechanism by which localized signaling at the branch tips enables cells to generate and transmit contractile forces to the surrounding matrix. Indeed, the role of contractile and tensile forces has been extensively studied in tissue development and morphogenesis,^[^ ^63^ ^]^ and our observations support a model in which actomyosin‐dependent contractions contribute to tissue extension,^[^ ^19^, ^64^ ^]^ which occurs in a non‐continuous manner.^[^ ^18^ ^]^ Previous measurements of bead displacements in 3D gels revealed that the volumetric expansion of multicellular spheroids pushes the surrounding matrix outward, while regions near extending cell fronts generate localized traction that pulls the matrix inward.^[^ ^65^ ^]^ This dynamic behavior is reminiscent of pulsed actin assembly during collective cell migration,^[^ ^66^, ^67^ ^]^ suggesting that temporally regulated force generation may be a conserved mechanism across diverse cellular and developmental processes.

We next evaluated how osmotic confinement regulates mammary branching and found that applying hyperosmotic pressure resulted in the inhibition of branch formation in slow stress‐relaxing matrices. As hyperosmotic stress reduces intracellular pressure, our work aligns with other studies that have found that morphogenetic processes rely on hydrostatic pressure to drive cellular expansion.^[^ ^68^ ^]^ In vivo, confinement can arise from changes in basement membrane composition, stiffness, or mechanical stability, and is known to play a critical role in shaping tissues by influencing cell behavior, tissue organization, and the coordination of intrinsic mechanical cues.^[^ ^11^ ^]^ In the context of mammary branching, it is known that the geometry and confinement of growing mammary tissues dictate their branching pattern.^[^ ^18^, ^22^ ^]^ Within broader epithelial morphogenetic processes, changes in osmotic gradients are correlated to lumenogenesis and epithelial cell proliferation,^[^ ^69^, ^70^ ^]^ and cells generate intracellular tension to drive the elongation of branches in the developing mouse lung.^[^ ^71^ ^]^ Although the application of hypoosmotic pressure did not enhance mammary branching, it is still possible that mammary branching is driven by changes in internal pressure, such as cell proliferation or other osmotic fluctuations. Hypoosmotic pressure was induced using previously established methods,^[^ ^34^, ^54^ ^]^ where the growth medium was diluted with DI water. However, we acknowledge that this dilution could introduce confounding effects on the cells. Previous work in the field has found that the release of osmotic pressure modulates cell proliferation and cell migration,^[^ ^72^, ^73^ ^]^ and this is consistent with our findings that releasing osmotic pressure is sufficient to drive mammary epithelial growth. Further, osmotic compression has been shown to suppress Rac1 activity and the protrusive remodeling of viscoplastic ECMs,^[^ ^74^ ^]^ further supporting the role of osmotic pressure and Rac1 as key regulators of mammary branching. Collectively, our findings contribute to a growing body of literature that cell volume regulation plays a key role in cellular processes such as mammary branching. Recent studies have shown that MSC volume expansion during cell spreading activates TRPV4 ion channels to enhance osteogenic differentiation,^[^ ^34^ ^]^ and cell volume regulation is driven by both chloride ion channels and ATP‐dependent processes.^[^ ^55^ ^]^ Further exploration of the role of TRP channels, mechanosensitive ion channels, and aquaporins in regulating and maintaining the pressure within mammary epithelium during branching could provide valuable insights into the mechanobiological processes underlying tissue morphogenesis.

Prior to this work, the effect of matrix viscoelasticity on branching morphogenesis generally, and on mammary branching specifically, remained unknown. Here, we highlight that matrix viscoelasticity is critical in regulating mammary branching morphogenesis. Importantly, we elucidate that mammary branching is mechanistically driven by dynamic extracellular matrix remodeling and intermittent epithelial contractions that are regulated via focal adhesion kinase and β1 integrin signaling. Looking forward, this framework can be expanded upon to further understand how branching morphogenesis is regulated by mechanical cues in other glandular epithelia, such as the lungs and kidneys. More broadly, these insights have implications for tissue engineering and regenerative medicine, where tuning of matrix mechanics could be leveraged to direct morphogenetic outcomes.

## 4 Experimental Section

### Alginate Preparation

Sodium alginate with a high molecular weight (FMC Biopolymer, Protanal LF 20/40, 280 kDa) was used to fabricate the slow stress‐relaxing matrices (*t*_1/2_ ≈ 1200 and 4000 s) and alginate with a low MW (UP VLVG, Pronova, 4200501 and UP LVG, Pronova, 4200001) was used to prepare fast stress‐relaxing matrices (*t*_1/2_ ≈ 100 and 800 s, respectively). Alginates were dissolved in deionized water (1% w/v) and then dialyzed with a tubing membrane (10 kDa MWCO) against deionized water for 3 days. The alginate solution was treated with activated charcoal, filtered (Sigma–Aldrich), sterile filtered (MilliporeSigma), frozen, lyophilized, and then dissolved in Dulbecco's Modified Eagle Medium: Nutrient Mixture F–12 (DMEM/F–12, ThermoFisher).

### Cell Culture and Spheroid Formation

MCF10A cells (RRID: CVCL_0598) were purchased from ATCC (CRL‐10317) beginning in 2020. Cells were tested and confirmed to be free of mycoplasma contamination. MCF10A cells were cultured in Dulbecco's Modified Eagle's Medium/Nutrient Mixture F–12 (DMEM/F–12, ThermoFisher), and supplemented with 5% horse serum (ThermoFisher), 20 ng mL^−1^ epidermal growth factor (Peprotech), 10 µg mL^−1^ insulin (Sigma), 0.5 µg mL^−1^ hydrocortisone (Sigma), 100 ng mL^−1^ cholera toxin (Sigma), and 1% penicillin/streptomycin (ThermoFisher), as previously described.^[^ ^75^ ^]^ MCF10A cells were passaged at 70% confluency, typically every three to four days, and the media was changed every 48 h. Cells were formed into MCF10A spheroids via the hanging‐drop method.^[^ ^76^ ^]^ Briefly, 6.8 µg mL^−1^ of rat tail collagen I solution (Advanced Biomatrix) was added to growth medium, and 15 µl droplets were pipetted onto the lid of a petri dish at a seeding density of 200 cells µL^−1^. The petri dish was inverted, and 5 mL of phosphate‐buffered saline (ThermoFisher) was added to the base of the petri dish to keep the spheroids hydrated. The petri dish was kept in a 5% CO_2_ humidified incubator at 37°C for 24–36 h to allow for spheroid formation.

### Alginate‐Collagen Hydrogel Fabrication and Spheroid Encapsulation

Hydrogels were fabricated using a syringe‐mixing method that has previously been described.^[^ ^52^ ^]^ First, warmed alginate was added into a 1 mL Luer lock syringe (Cole‐Palmer), avoiding the creation of air bubbles.^[^ ^52^ ^]^ Extra DMEM/F–12 (ThermoFisher) was added such that all matrices had a final alginate concentration of 0.5% w/v, except for the fabrication of the slow stress‐relaxing matrix (*t*_1/2_ ≈ 4000 s), which had a final concentration of 1.5% w/v. Next, calcium sulfate dihydrate (Sigma‐Aldrich) was used to form ionic crosslinks with alginate chains. Calcium sulfate was dissolved in deionized water, autoclaved, and subsequently added to a separate 1 mL Luer lock syringe (Cole‐Palmer) such that the final concentration was 2 mm. Rat tail collagen I (Advanced Biomatrix) was kept on ice and added to the syringe such that the final concentration was 2 mg mL^−1^. The collagen was immediately neutralized with 10 mm sodium hydroxide (NaOH). The two syringes were then coupled with a Luer lock (Cole‐Palmer) and rapidly mixed with 20 pumps on the syringe handles. For mechanical characterization tests, these substrates were deposited directly onto the parallel plate of the rheometer. For cell culture experiments, these gels were deposited directly into a sterile 48 well plate (Fisher Scientific) after spheroids were counted and added into the syringe with warmed alginate. The well plate was then immediately transferred to a 37°C incubator, and the gel was allowed to polymerize for 1 h prior to adding cell‐culture medium. The culture medium was changed every two days.

To fabricate covalently‐crosslinked tissue transglutaminase (tTG)‐collagen matrices, tTG from guinea pig liver (Sigma‐Aldrich) was dissolved in 50 mm Tris Buffer (pH 7.4) and treated for 10 min at room temperature with dithiothreitol (DTT) solution such that the final concentration of DTT was 2 mm and the final concentration of tTG was either 20 or 40 µg mL^−1^. Then, 5 mm CaCl_2_ was added to activate the tTG, along with collagen I and sodium hydroxide (10 mm) to neutralize the collagen. The final concentration of collagen was fixed at 2 mg mL^−1^ for all concentrations of tTG by adding the appropriate volume of DMEM/F–12. For collagen‐only matrices, Rat tail collagen I (Advanced Biomatrix) was neutralized with 10 mm of sodium hydroxide, and DMEM/F–12 (ThermoFisher) was added such that the final concentration of collagen was 2 mg mL^−1^.

### Mechanical Characterization of Alginate‐Collagen IPNs

Mechanical properties of the hydrogel were assessed using in situ testing with an Anton Paar MCR 502 stress‐controlled rheometer. Briefly, alginate hydrogels with low molecular weight alginate and high molecular weight alginate were fabricated, as described above. A 25 mm plate was lowered onto the polymer solution until the rheometer registered a non‐negative axial force immediately after crosslinking commenced. Gels were maintained at 37 °C. A thin layer of mineral oil was then pipetted around the hydrogel to ensure the gels would not dehydrate during mechanical measurements. Once the storage modulus reached an equilibrium value, a stress relaxation test was performed. While the strain (10%) was held constant, stress was recorded over time. The stress relaxation time was defined as the time taken for the maximum stress to relax to half of its initial value. The storage and loss modulus of the hydrogel were measured using a time sweep test (1 Hz, 1% strain). From this data, the complex modulus (Equation 1) and Young's modulus were calculated (Equation 2). The complex shear modulus (G*) describes the material's response to shear stress, whereas the Young's modulus (E) describes the material's response to uniaxial stress. A Poisson's ratio (𝑣) was assumed to be 0.5.^[^ ^52^ ^]^

(1) $$G*={\left({G^{\prime }}^{2}+{G^{\prime \prime }}^{2}\right)}^{1/2}$$

(2) $$E=2\left(1+v\right)G*$$

### Microscopy

All confocal images were collected using a laser scanning confocal microscope (Leica SP8). All brightfield images were collected using an Olympus IX50 – S8F2 inverted microscope. In live‐cell time‐lapse imaging, a Leica SP8 confocal microscope was utilized and equipped with an Okolab incubation chamber (37°C and 5% CO_2_). MCF10A spheroids were encapsulated with 0.2 µm dark red carboxylate‐modified fluorescent microspheres (Molecular Probes) at a density of 1.6 × 10^12^ beads per mL and dispensed into chambered cover glasses (ThermoFisher). Spheroids were imaged every 10 min overnight using a Leica HC PL Fluotar 10x/0.30 objective. To visualize the collagen network of the alginate‐collagen IPNs, spheroids were first fixed on day four using 4% paraformaldehyde for 45 min at 37°C. The gels were then washed three times with Dulbecco's phosphate‐buffered saline with calcium (cPBS) for 20 min each time and then transferred to a glass cover slip. After this, a Leica 25x immersion objective was utilized to perform confocal reflectance microscopy.^[^ ^77^ ^]^

### Immunofluorescence Staining of Fixed Cells

For the phosphorylated FAK and β1 integrin immunofluorescence analysis, all spheroids were fixed and washed as previously described. After this, they were left overnight in a 30% w/v sucrose in cPBS solution to dehydrate the gels. The following day, the gels were transferred to a solution of 50% w/v OCT and 50% w/v of the 30% w/v sucrose‐cPBS solution for 6 h to allow the OCT to penetrate the gel. Gels were then transferred into cryomolds with OCT and allowed to freeze on dry ice before being transferred to −20°C. The samples were then sectioned with a cryostat (Leica) to 60 µm sections and transferred to glass Superfrost Plus slides (Thermofisher). The sections were then washed with cPBS and then incubated in a blocking buffer for 1 h to minimize non‐specific staining. Samples were then incubated with primary antibodies overnight, washed with cPBS, and then incubated with secondary antibodies for 1 h. Primary antibodies used: β1 integrin *1:200* (Thermofisher, 14‐0299‐82), FAK‐P‐Y397 *1:500* (Thermofisher, 700255). Secondary antibodies used: Alexa Fluor Goat Anti‐Ms IgG1 *1:1000*, 647 (Thermofisher, A21240), Alexa Fluor 488 Goat Anti‐Rb IgG1 *1:1000* (Thermofisher, A11008). Fluorescent dyes used: Hoechst 33342 *1:500* (ThermoFisher, 62249) and Octadecyl Rhodamine B (R18) *1:1000* (ThermoFisher, O246).

### Image Analysis

Metrics describing spheroid area and circularity were quantified using Image J (version 1.51) software. In Image J, circularity was defined as (4π x area) / perimeter,^[^ ^2^ ^]^ which ranges from zero to one, where one represents a perfect circle. Branch length was manually quantified as the length from the boundary between the branch and the spheroid to the tip of the branch. Feature sizes less than 40 µm were excluded from analysis. The total number of branches, including all primary, secondary, and tertiary branches, was manually counted.

Immunofluorescence intensity data were analyzed via a custom pipeline developed in *CellProfiler* v4.2.6, an open‐source segmentation algorithm designed to automate cellular phenotypic analysis.^[^ ^78^ ^]^ To determine regional variations in immunostaining intensity, objects were first identified via the *IdentifyPrimaryObjects* module, and then subsequently, a *MeasureObjectIntensityDistribution* module was employed to enable automatic and equidistant segmentation and analysis of the tip, body, and branch regions of the spheroids. This pipeline was conducted separately for all β1 integrin, phosphorylated FAK, and nuclei images. All phosphorylated FAK and β1 integrin intensity values were normalized against the intensity of the nuclei stain in each region.

To quantify the orientation of collagen fibers, an open‐source quantitative tool was employed.^[^ ^79^ ^]^ Briefly, images were imported into CurveAlign (4.0), and the “CT” fiber analysis method via “Tiff Boundary” was selected. Branches were manually segmented, and ROIs were incorporated to delineate collagen fiber orientations along the branch axis and adjacent to the branch axis. This method of analysis has been previously reported.^[^ ^40^ ^]^ The program generated a CSV file with all the orientation angles it tracked. To eliminate bias from the total number of collagen fibers counted, the data were binned and then divided by the total number of fibers found in each ROI. Polar plots were generated via a custom MATLAB script.

### Matrix Displacement Analysis

To generate the matrix displacement maps, bead channel images were generated into a stack and corrected for drift using the ImageJ plugin Linear Stack Alignment with SIFT. Then, the ImageJ particle image velocimetry (PIV) plugin was implemented using a cross‐correlation window of 64 pixels and 32 pixels to yield 16‐pixel displacement vectors, per previously established protocols.^[^ ^80^, ^81^ ^]^ This PIV analysis generated a vector field of matrix displacements, which was then imported into the ImageJ PIV plot plugin to generate magnitude and vector displacement plots, with a manually set vector scale threshold. Brightfield images of the spheroid were overlaid onto the matrix displacement field using ImageJ.

For quantification of bead displacement, Imaris 10.2.0 was used. The Track Spots algorithm was selected to track the identified spots as objects over several sequential frames in the time series dataset. ROIs were used to delineate regional differences in quantifications. The reference frame was manually oriented so that the *y*‐axis was aligned toward the spheroid. The Autoregressive Motion algorithm was implemented to predict the location and direction of the spot based on the previous frame. The hyperparameters were selected to include a maximum distance of 5 µm to reduce errors in tracking and to ensure the predicted future position of a spot was reliably based on its previous position. Additionally, the max gap parameter was set to 3 µm to reduce the risk of incorrect spot connections across frames. The position data was exported as a. csv file, and a custom MATLAB script was written to group track IDs and quantify the aggregated displacement over time. Additionally, the script calculated the displacement delta over time for each track. The average of all beads in at least 15 spheroids across 3 replicates was analyzed to quantify the mean displacement along the branching axis.

### Inhibition Studies

Pharmacological inhibitors were added to the growth medium 1 h after hydrogel gelation. Media with inhibitors was changed every two days. The inhibitors used were: 50 µm NSC 23766 (Tocris Bioscience, Rac1 inhibitor), 50 µM Blebbistatin (Cayman Chemical, non‐muscle myosin II inhibitor), and 10 µm PF 573228 (Cayman Chemical, FAK inhibitor). Brightfield images were collected on day seven of culture and morphologically analyzed via methods previously described.

### Modulation of Hyperosmotic and Hypoosmotic Pressure

Hyperosmotic stress was induced by adding various concentrations of PEG 400 (TCI America) to the culture medium at final concentrations of 0% wt/vol (0 mOsm L^−1^), 1.5% wt/vol (37.5 mOsm L^−1^) and 3% wt/vol (75 mOsm L^−1^), which correspond respectively to osmotic pressure increases of 0, 92, and 197 kPa. Hypoosmotic stress was induced by replacing 20% or 40% of the culture medium with deionized water. Osmotic pressures were calculated via an empirically derived formula, where c represents the wt/vol concentration of PEG 400 in media (Equation 3).^[^ ^82^, ^83^ ^]^ In osmotic pressure experiments where the culture medium remained unchanged throughout the experiment, hypertonic and hypotonic media were added on day zero, 1 h after matrix gelation. Brightfield images of spheroids were collected on day seven and analyzed using methods previously described. In dynamic osmotic pressure experiments, hypertonic media were either: 1) added on day zero and replaced with standard growth medium on day four, or 2) added on day four following four days of culture in standard growth medium. Brightfield images were taken on days zero, four, and seven, and images were analyzed via methods previously described.

(3) $$y\left(\mathrm{atm}\right)=.00002c^{4}.-0007c^{3}+.0311c^{2}+.5596c$$

### Statistical Analysis and Reproducibility

Statistical analyses were performed using GraphPad Prism 9.1.0. Data was analyzed using Student's *t*‐tests for two groups. For more than two groups, data was analyzed using either a one‐way ANOVA or a Kruskal–Wallis test. Post hoc multiple comparisons were conducted using Tukey's, Šídák's, or Dunn's tests, as appropriate. Statistical significance was defined as ^****^ *p* < 0.0001, ^***^ *p* < 0.001, ^**^ *p* < 0.01, ^*^ *p* < 0.05, and n.s. = not significant. Data were presented as mean ± SD from three independent biological replicates, with *n* = 5–15 images or videos per replicate.

## Conflict of Interest

The authors declare no conflict of interest.

## Author Contributions

D.I.W. performed conceptualization, methodology, investigation, visualization, formal analysis, writing, editing. J.W.M. performed methodology, investigation, formal analysis, editing. A.S. performed conceptualization, methodology, investigation, editing. R.S.S. performed conceptualization, methodology, investigation, visualization, formal analysis, supervision, writing, editing.

## Supporting information



Supporting Information

Supplemental Video 1

Supplemental Video 2

## Data Availability

The data that support the findings of this study are available from the corresponding author upon reasonable request.
