# Supplementary material for: Extracellular Matrix Viscoelasticity Regulates Mammary Branching Morphogenesis
Source: Adv Sci (Weinh). 2025 Nov 10;13(3):e12873. doi: 10.1002/advs.202512873 (PMC12806548; doi:10.1002/advs.202512873)
Supplement: Supplementary file 1 — Supporting Information [file ADVS-13-e12873-s002.docx]

**Supplementary Video 1:** **Representative MCF10A spheroid in a fast stress relaxing matrix (t_1/2_ ≈ 100 s) undergoing isotropic expansion.** Time-lapse imaging shows brightfield channel overlaid with the fluorescent bead channel (green), taken 48 hours after encapsulation. Frames were acquired every 10 minutes for over 16 hours.

**Supplementary Video 2:** **Representative MCF10A spheroid in a slow stress relaxing matrix (t_1/2_ ≈ 1200 s) undergoing branching morphogenesis.** Time-lapse imaging shows brightfield merged with a fluorescent bead channel (green), taken 48 hours after encapsulation. Frames are taken every 10 minutes for over 16 hours.


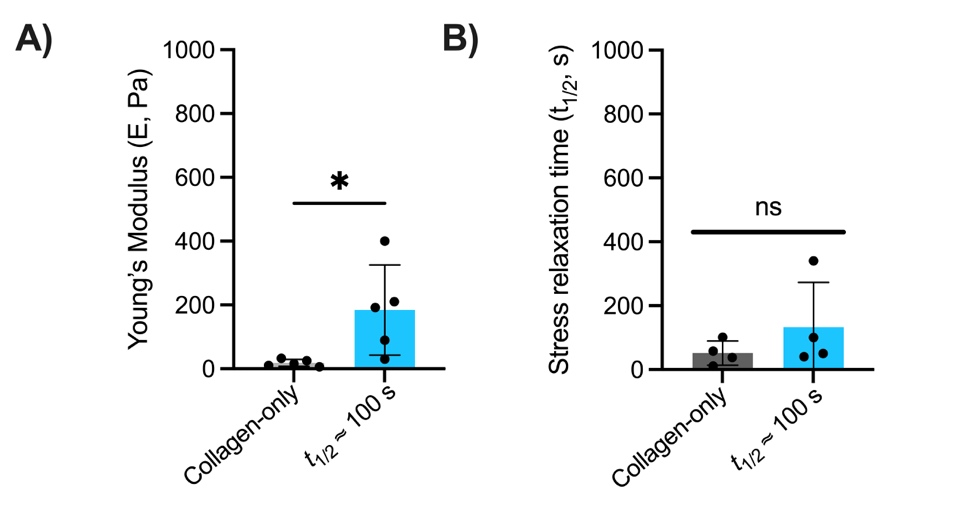


**Supplementary Figure 1: Mechanical characterization of collagen-only gels compared to fast relaxing alginate-collagen gels.** (a) Time sweep reveals collagen-only gels are significantly softer than alginate-collagen gels. (b) Both collagen-only and alginate-collagen gels exhibit fast stress relaxation. p values are calculated by a Student’s *t-*test. *p < .05 and n.s. = no significant difference. All data are represented as mean +/- SD.


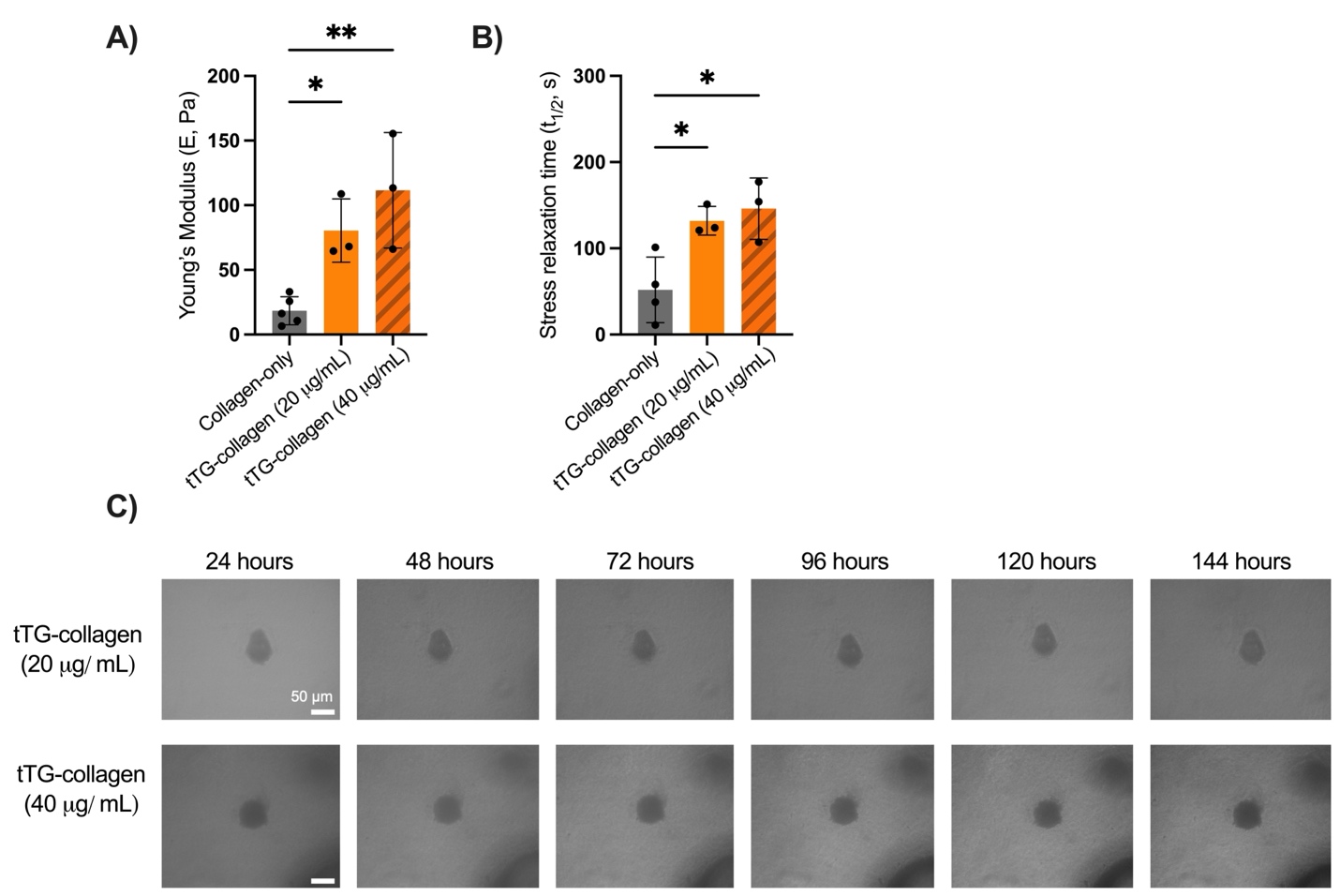


**Supplementary Figure 2: Covalent crosslinking of collagen gels with tissue transglutaminase (tTG) does not promote mammary branching.** (a) Collagen-only gels are significantly softer than tTG-collagen gels. (b) Stress-relaxation tests on collagen-only gels and tTG-crosslinked collagen matrices. (c) Brightfield imaging of MCF10A spheroids cultured in 20 or 40 $\mu$g/mL tTG-collagen gels over 6 days shows limited growth and no branching. Statistical significance is calculated by a one-way ANOVA and Brown-Forsythe test: **p < .01 and *p < .05. All data are represented as mean +/- SD.

**
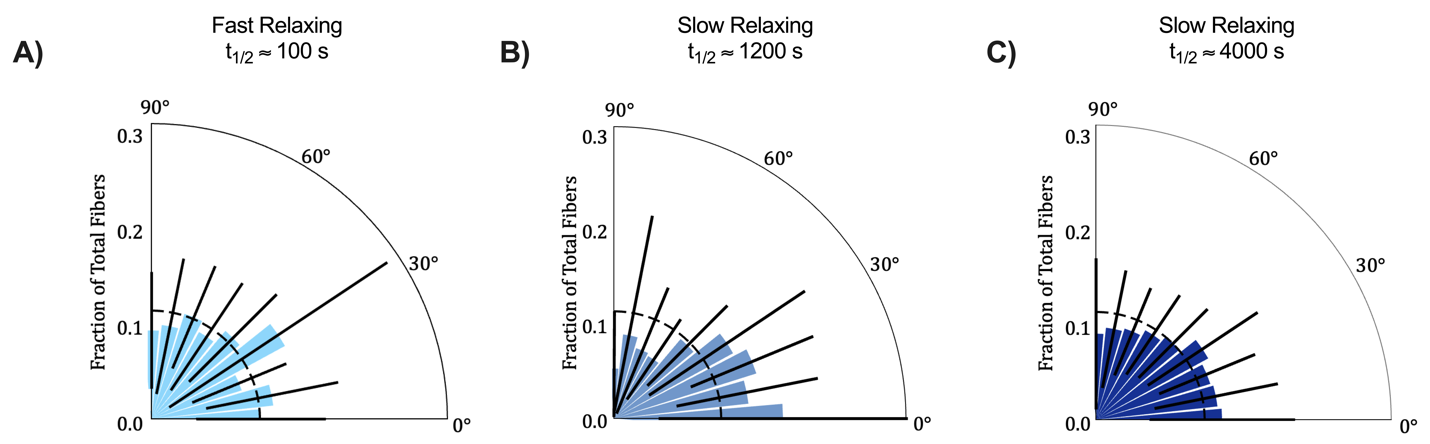
**

**Supplementary Figure 3: Collagen fiber alignment transverse to branching axis is randomly aligned regardless of matrix stress relaxation.** (a) Quantification of relative orientation angle transverse to the branching axis in the t_1/2_ ≈ 100 s matrix, (b) t_1/2_ ≈ 1200 s matrix, and (c) t_1/2_ ≈ 4000 s matrix. Collagen alignment was quantified via *CurveAlign*. n = 9 images from 3 hydrogels per condition. Black bars represent SD for each bin.


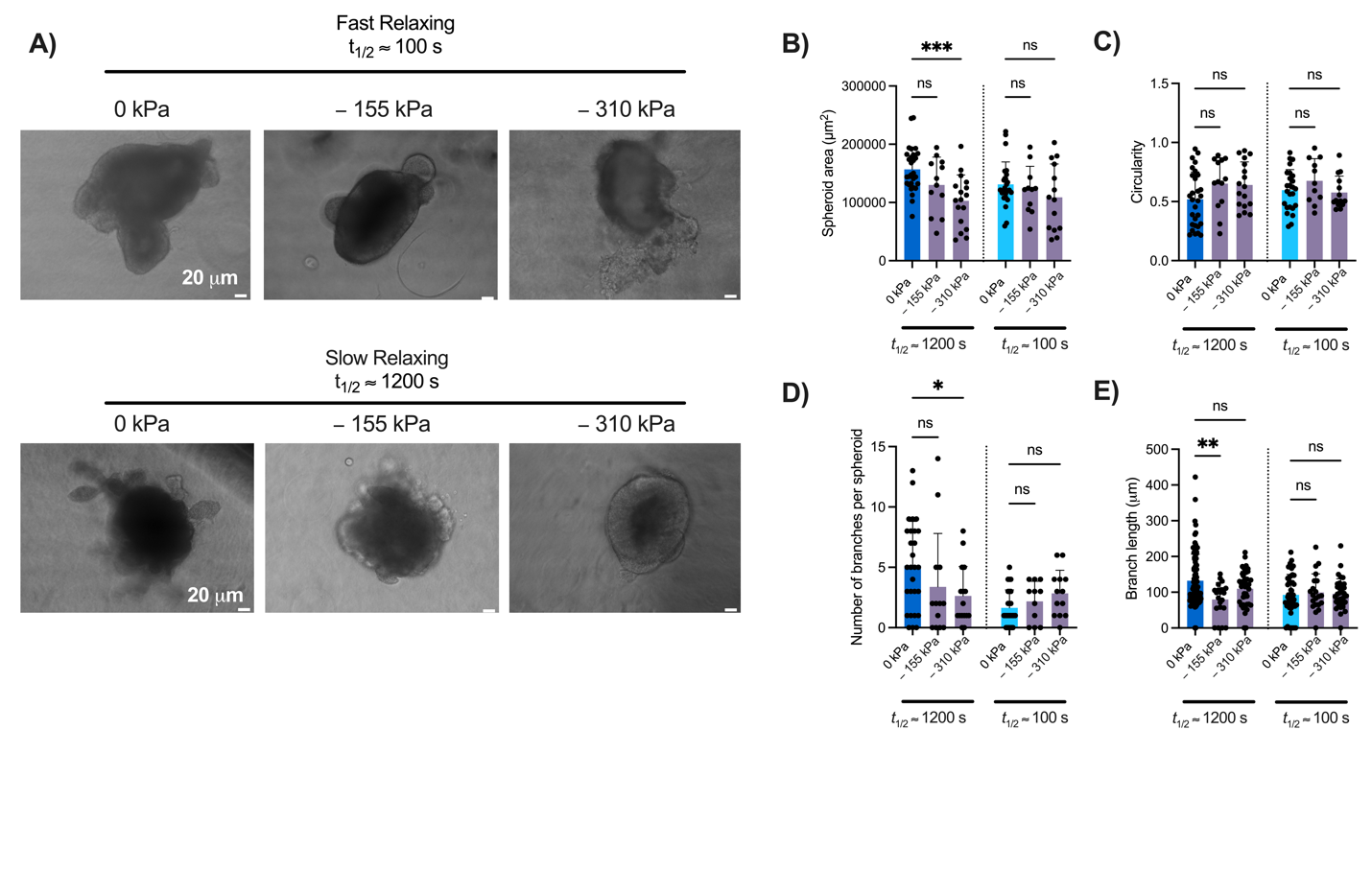


**Supplementary Figure 4: Hypoosmotic stress does not promote mammary branching.** (a) Brightfield images of MCF10A spheroids in various stress relaxing conditions (t_1/2_ ≈ 100 s, t_1/2_ ≈ 1200 s) under different hypoosmotic stresses following 7 days of culture. (b) Quantification of MCF10A cross-sectional spheroid area under varied hypoosmotic pressure for 7 days. (c) Circularity is not significantly different in slow or fast stress relaxing matrices when spheroids are subjected to hypotonic media. (d) Total branches produced per spheroid decreases when exposed to – 310 kPa in slow stress relaxing matrices, and there is no significant difference in fast stress relaxing matrices. (e) Quantification of branch length demonstrates that spheroids branch to a lesser extent in slow stress relaxing matrices when exposed to – 155 kPa. Statistical significance calculated using one-way ANOVA with Šídák’s multiple comparison tests: ***p< .001, **p<.01, *p<.05 and n.s. = not significant. n = 5-15 images from 3 independent experiments. All data are represented as mean +/- SD.


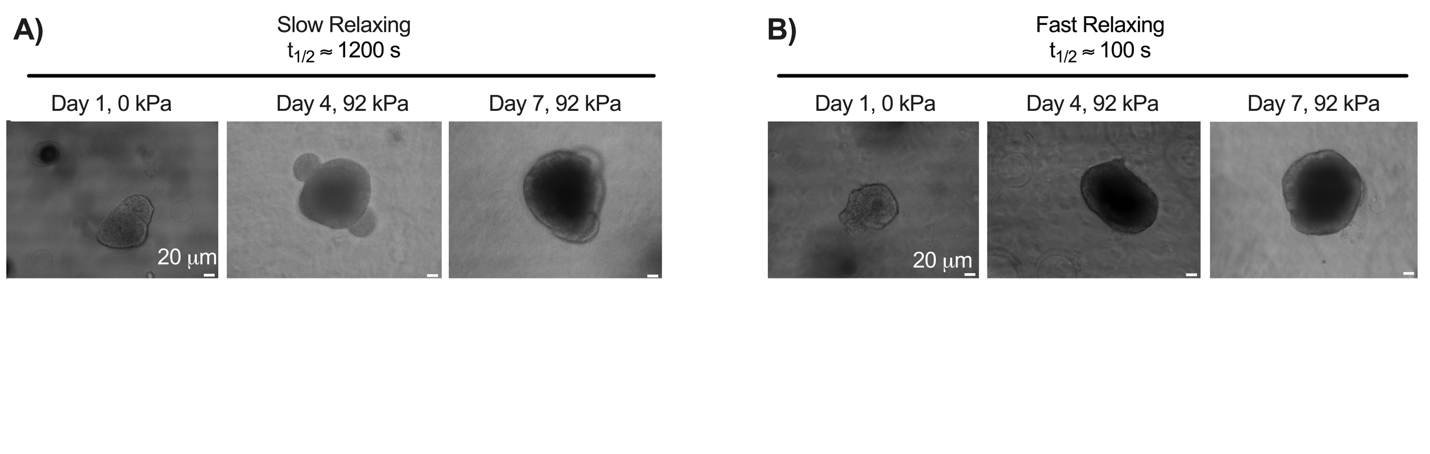


**Supplementary Figure 5: Hyperosmotic stress dynamically impedes mammary branching.** (a) Representative brightfield images of MCF10A spheroids in slow stress relaxing conditions (t_1/2_ ≈ 1200 s) and (b) fast stress relaxing conditions (t_1/2_ ≈ 100 s) after osmotic pressure (92 kPa) was applied on Day 4 in culture.


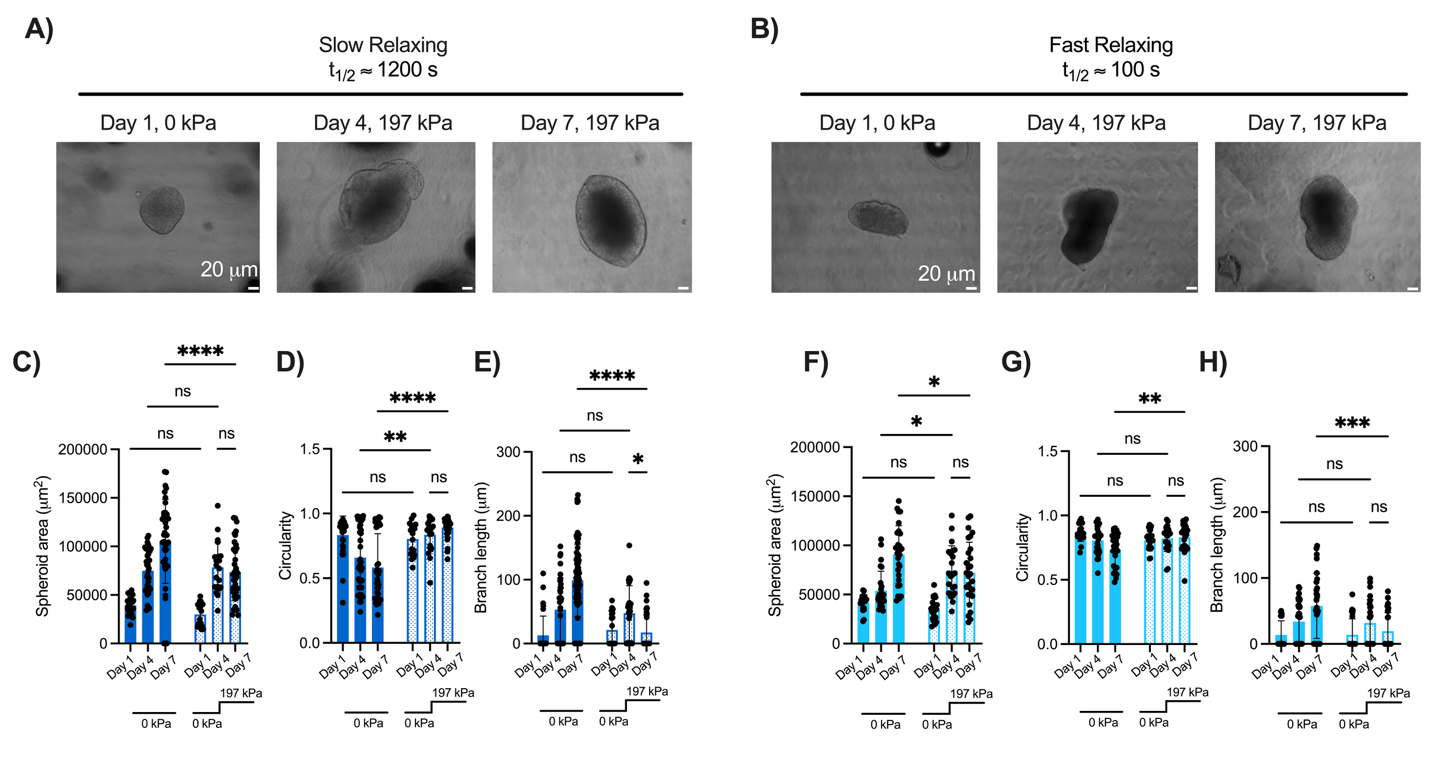


**Supplementary Figure 6: Dynamic changes to higher hyperosmotic stresses impair mammary epithelial branch formation and growth in slow stress relaxing matrices.** (a) Representative brightfield images of MCF10A spheroids in slow stress relaxing conditions (t_1/2_ ≈ 1200 s) and (b) fast stress relaxing conditions (t_1/2_ ≈ 100 s) after higher osmotic pressure ($\triangle P=$197 kPa) was applied on Day 4 in culture. (c) MCF10A spheroids are unable to resume growth in slow stress relaxing matrices and in (f) fast stress relaxing matrices after osmotic pressure has been applied for 4 days. (d, e) Circularity is enhanced and branch length decreases when subjected to higher osmotic stresses in both slow and (g, h) fast stress relaxing conditions. Statistical significance calculated using one-way ANOVA with Šídák’s multiple comparison tests: ****p < .0001, ***p < .001, **p<.01, *p<.05 and n.s. = not significant. n = 5-15 images from 3 independent experiments. All data are represented as mean +/- SD.


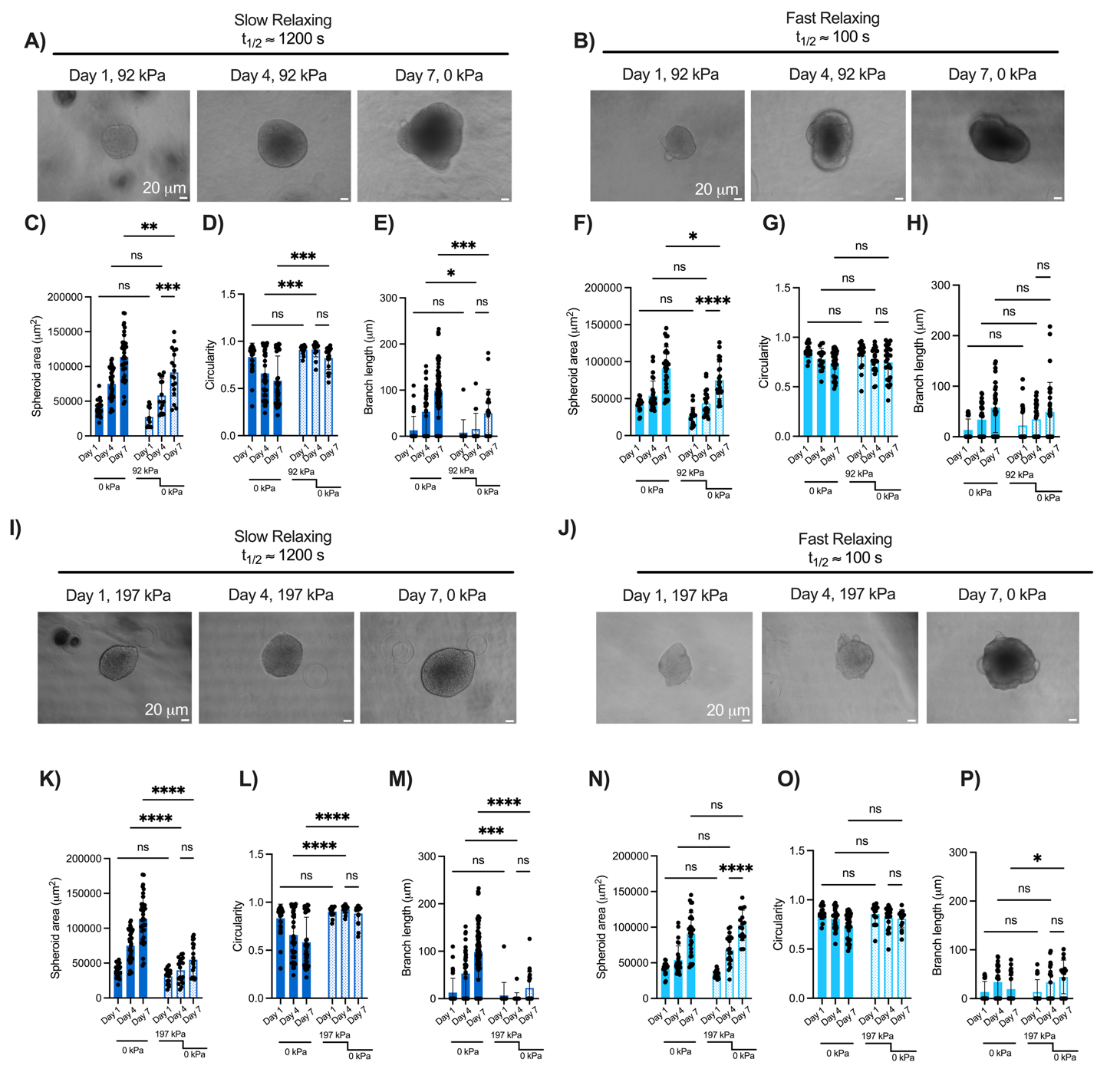


**Supplementary Figure 7: MCF10A cells are able to resume growth, but not branching, following removal of hyperosmotic stress**. (a) MCF10A spheroids are able to resume growth in slow stress relaxing matrices and (b) fast stress relaxing matrices after osmotic pressure ($\triangle P=$92 kPa) has been applied for 4 days. (c, f) Quantification of MCF10A spheroid area demonstrates MCF10A spheroids can resume growth in both slow and fast stress relaxing matrices after osmotic pressure is alleviated. (d, e) MCF10A spheroids are unable to resume significant branching after being subjected to osmotic pressure in slow stress relaxing matrices, and there are no significant changes in circularity. (g, h) There is no significant difference in circularity or branch length once hyperosmotic pressures have been removed from fast stress relaxing matrices. (i) Representative brightfield images of MCF10A spheroids in slow stress relaxing conditions (t_1/2_ ≈ 1200 s) and (j) fast stress relaxing conditions (t_1/2_ ≈ 100 s) after higher osmotic pressure ($\triangle P=$197 kPa) was released on Day 4 in culture. (k) MCF10A spheroid growth in slow stress relaxing matrices was halted after osmotic pressure was applied and released, and this was accompanied by (l) increased circularity and (m) decreased branch length compared to the control. (n) In fast stress relaxing matrices, there is no significant difference in cross-sectional area nor (o) circularity after osmotic pressure has been applied and released. (p) MCF10A spheroids in fast stress relaxing matrices can resume branching following the release of osmotic stress. Statistical significance calculated using one-way ANOVA with Šídák’s multiple comparison tests: ****p < .0001, ***p < .001, **p<.01, *p<.05 and n.s. = not significant. n = 5-15 images from 3 independent experiments. All data are represented as mean +/- SD.
